# Effects of incorporation of silver and titanium nanoparticles on feldspathic ceramic toughness

**DOI:** 10.15171/joddd.2019.015

**Published:** 2019-08-14

**Authors:** Vasudevan Karthikeyan, Naveen Gopi Chander, Jetti Ramesh Reddy, Balasubramanium Muthukumar

**Affiliations:** ^1^Senior Lecturer, Department of Prosthodontics, Sri Ramaswami Memorial Dental College, Ramapuram, Chennai, India; ^2^Professor, Department of Prosthodontics, Sri Ramaswami Memorial Dental College, Ramapuram, Chennai, India; ^3^Reader, Department of Prosthodontics, Sri Ramaswami Memorial Dental College, Ramapuram, Chennai, India; ^4^Department of Prosthodontics, Sri Ramaswami Memorial Dental College, Ramapuram, Chennai, India

**Keywords:** Nano-reinforcement, feldspathic ceramic toughness, silver-reinforced ceramics, titanium reinforced ceramics

## Abstract

***Background.*** Porcelain is brittle and in many situations it requires replacement in fractured or chipped restorations. The prospects exist in improving the strength of feldspathic porcelain to widen its applications. This study evaluated the fracture toughness of feldspathic porcelain after incorporation of silver and titanium nanoparticles at varying concentrations (0.5 wt%, 1 wt%, 1.5 wt% and 2 wt%).

***Methods.*** Test specimen dimensions were standardized to ASTM C1421–16 standards and a three-point flexure test was carried out to evaluate fracture toughness. A total of 330 samples were fabricated and broadly divided into three groups. Group I (titanium nanoparticles) and group II (silver nanoparticles) were further subdivided into 5 groups (0 wt%, 0.5 wt%, 1 wt%, 1.5 wt% and 2 wt%) for fracture toughness analysis, with each group consisting of 30 samples. Group III contained the superior concentration of both titanium and silver nanoparticles. The fracture toughness (KIC) was calculated using indentation fracture method and microstructure observations were made using scanning electron microscopy. The KIC values were compared and evaluated using one-way ANOVA.

***Results.*** Data were analyzed using one-way ANOVA and Tukey’s HSD post hoc test multiple comparisons. The mean values of group I, group II and group III were 1.949 MPa.m1/2, 2.002 MPa.m1/2 and 1.330 MPa.m1/2 , respectively.

***Conclusion.*** The results revealed that the samples reinforced with titanium and silver nanoparticles showed significant increases in fracture toughness. The blending of superior concentration of both titanium and silver nanoparticles decreased fracture resistance.

## Introduction

Dental porcelain has been widely used as a fixed dental prosthesis material (FDP). It provides excellent esthetic results, simulating natural teeth but it has functional limitations due to its strength.^1^ The rationale, use, development and selection of various ceramic materials has been enumerated in the literature.^2,3^

Advanced ceramics have greater advantages  and few  limitations such as esthetics, additional layering, high cost of equipment and less versatility.^4^ Moreover, the conventional ceramic FDPs are still in use (75%) and widely employed for layering  ceramic copings.^5^  Limitations of  chipping or fracture of restorations are  reported due to regular ceramics.^6^ The advancement of technology and newer available nano-materials provide the  scope of improving the strength of the materials to broaden their applications

The methods to improve the mechanical properties of feldspathic porcelain involves reinforcement with other materials.^7^ Porcelain materials were reinforced with leucite crystals and fibers in the earlier years to nanoparticles in recent days.^8-10^ Various nanoparticles  such as silver oxide (AgO),^11^ zirconia (ZrO_2_),^12^ alumina (Al_2_O_3_),^14^ hydroxyapatite (HA),^11^   titanium dioxide (TiO_2_)^13^ and platinum (Pt)^15^ have been  tried in different proportions   to improve the properties of feldspathic porcelain.^11-15^ Uno et al,^1^ Mohsen et al,^11^ Esparza-Vázquez et al,^13^ Fujieda et al^15^   and Siegel et al^16^ have determined the fracture toughness with various nanoparticles. The results from these studies were supportive but still prospects exist in improving the properties of feldspathic porcelain. The properties of the material can be enhanced by incorporating one or two nanoparticles to obtain superior benefits. The literature has limited studies on the incorporation of two nanoparticles into dental porcelain.  In this study, silver and titanium nanoparticles were incorporated into porcelain, and the effect of these nanoparticles on the fracture toughness of the porcelain was evaluated. The superior concentrations of each group of nanoparticles were combined to evaluate the strength of feldspathic porcelain. The null hypothesis was that incorporation of silver and titanium nanoparticles has no effect on the fracture toughness and Vickers hardness of porcelain.

## Methods

ASTM C1421–16 guidelines were followed to fabricate the test specimens.^16^  An alloy mold that can withstand higher temperature for multiple firings was fabricated for the required specimen dimensions.  The dimension of the mold was 30×3×4 mm with 0.2-mm taper, for ease of specimen removal. The designing of the mold was carried out with CNC (Computer Numerical Control, Zen Machine Tools). The dimensions of the specimens were rechecked with a digital Vernier caliper (Insize Vernier Caliper 150 mm/6”- Widescreen Digital Electronic).

100-nm silver oxide and 25-nm titanium dioxide nanoparticles were procured from an external source (Sigma-Aldrich Co. LLC). The material contained 99.5% of trace metals and polyvinyl pyrrolidone (PVP) as dispersant. The nanoparticles were measured using a digital weighing machine (AC22-300 - Accurate Electronics Precision Scale Weighing Machine) for various concentrations (0.5 gr, 1 gr, 1.5 gr, 2 gr) and transferred to the dentin body of feldspathic porcelain (Noritake Super (NS) Porcelain EX-3 (Dentin Body; Kuraray Noritake Dental Inc.). Planetary ball milling was carried out for blending the nanomaterials with porcelain.

The blended ceramic nanoparticles were mixed with the universal modeling liquid, shaped on the mold, and the specimens were fabricated. A thin layer of ceramic separating medium was used for the ease of specimen removal after completion of the firing cycle. The required amount of mixture was loaded in the mold and sintered in a ceramic furnace (Ivoclar Vivadent Programat P310). The sintering of porcelain was carried out at 870ºC with a heating rate of about 60ºC/minute; the vacuum was released at about 869ºC and maintained for 15 minutes. The sintered specimens were removed by tapping the extruders inserted at the base of the mold. The specimens were retrieved from the mold, checked, trimmed and glazed.

**Figure 1:**
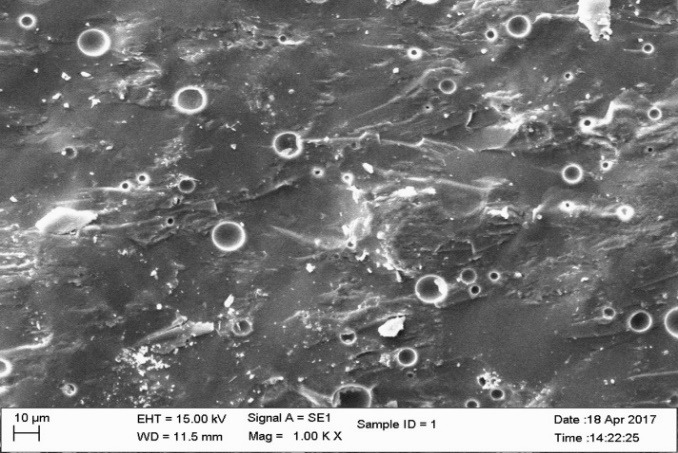
SEM analysis of non-reinforced conventional ceramics.

**Figure 2:**
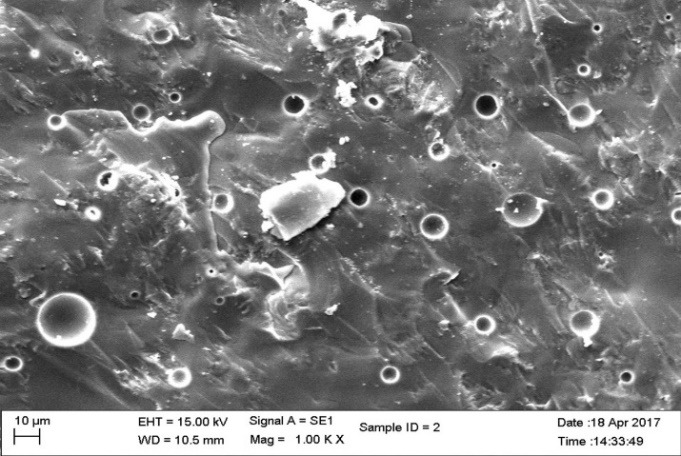
SEM analysis of 2 wt% of nano-titanium-reinforced samples.

**Figure 3:**
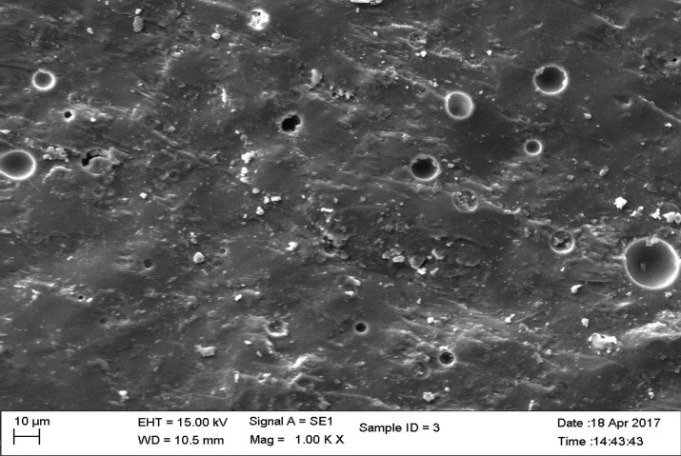
SEM analysis of 2 wt% of nano-silver-reinforced samples.

**Figure 4:**
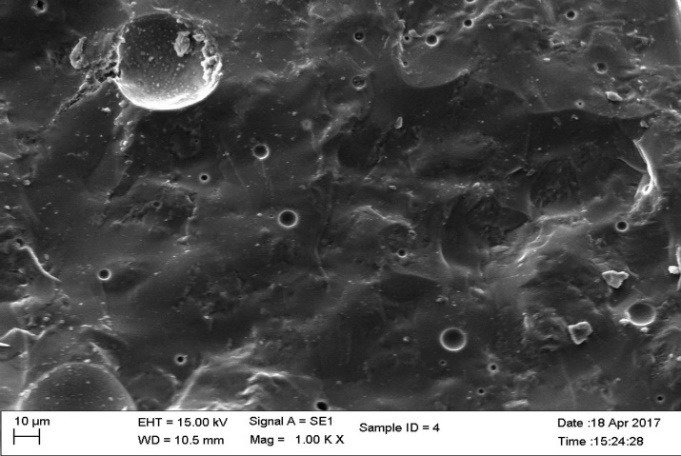
SEM analysis of blending of superior concentration of titanium and silver reinforced samples.

Three groups of material were fabricated for fracture toughness test.  Groups 1 and 2 were subdivided into 5 subgroups according to percentage of nanoparticles. The concentration of the highest strength of groups 1 and 2 were used to fabricate group 3 specimens. The composition, content and type of nanoparticles in each group are listed in Table 2. A total of 330 samples were fabricated. The finished specimens were evaluated for fracture toughness by 3-point bending test and scanning electron microscope (SEM) observations were made (Figures 1 to 4). The KIC values were compared and evaluated by one-way ANOVA.

## Results

The results were normally distributed. The mean fracture toughness values of the control and reinforcement group are listed in Tables 1 to 3. The mean fracture toughness of the control group was 1.809 MPa.m^1/2^. The fracture toughness of groups I, II and III were 1.949 MPa.m^1/2^, 2.002 MPa.m^1/2^ and 1.330 MPa.m^1/2^, respectively, with the highest concentration of nanoparticles. Data were analyzed using one-way ANOVA between groups and Tukey HSD post hoc tests for multiple comparisons between the groups, represented in Table 4.

**Table 1:** Group I with various concentrations of titanium nanoparticles

**Group 1**	**Titanium nanoparticle concentration**	**Samples**
**1a**	-	30
**1b**	0.5 gr	30
**1c**	1 gr	30
**1d**	1.5 gr	30
**1e**	2 gr	30

**Table 2:** Group II with various concentrations of silver nanoparticles

**Group 2**	**Silver nanoparticle concentration**	**Samples**
**2a**	-	30
**2b**	0.5 gr	30
**2c**	1 gr	30
**2d**	1.5 gr	30
**2e**	2 gr	30

**Table 3:** Group III with superior concentrations of both titanium and silver nanoparticles

** Group 3**	**Blending the better of both Group 1 and Group 2**	**30**

**Table 4:** Comparison of mean fracture toughness between groups using one-way ANOVA

**Group**	**N**	**Mean**	**Std. Dev**	**F-Value**	**P-value**
**Control**	**60**	**1.8087**	**0.05619**	**344.560**	**<0.001**
**Titanium**	**120**	**1.9493**	**0.07436**		
**Silver**	**120**	**2.0023**	**0.14560**		
**Silver & Titanium**	**30**	**1.3303**	**0.03978**		

**Table 5:** Multiple comparisons between group I, II and III using Tukey HSD post hoc tests

**Group**		**Mean Difference**	**Std. Err.**	**P-value**
**Control**	**Titanium**	-.14058	0.02161	<0.001
	**Silver**	-.19358	0.02161	<0.001
	**Silver and Titanium**	.47833	0.02734	<0.001
**Titanium**	**Silver**	-.05300	0.01367	0.001
	**Silver and Titanium**	.61892	0.02161	<0.001
**Silver**	**Silver and Titanium**	.67192	0.02161	<0.001

Statistically significant differences were seen between the groups at P<0.001. SEM images (Figures 1 to 4) were obtained to observe the distribution of particles.

## Discussion

This study investigated the fracture toughness of feldspathic ceramic after incorporation of silver and titanium nanoparticles at various compositions (0 wt%, 0.5 wt%, 1 wt%, 1.5 wt%, 2 wt%) and by blending the superior concentration of both silver and titanium nanoparticles obtained from reinforcements. The highest concentrations of test samples were limited to 2 wt% to aid in standardization and to reduce the toxicity of silver nanoparticles.^17^  The null hypothesis was that the conventional feldspathic ceramic has no significant difference in fracture toughness after incorporation of silver and titanium nanoparticles. By infusing the superior concentration of silver (2 wt%) and titanium (2 wt%) nanoparticles the fracture toughness of conventional feldspathic ceramic was not significantly different when compared to the individual blending of silver and titanium nanoparticles. The altered conclusion is that conventional feldspathic porcelain has significant fracture toughness after incorporation of silver and titanium nanoparticles. With the infusion of the superior concentration of both silver and titanium nanoparticles to the conventional feldspathic porcelain there was a significant difference in the fracture toughness. The null hypothesis was not accepted; incorporation of silver and titanium nanoparticles significantly increased the fracture toughness and was confirmed with NS porcelain. The null hypothesis was accepted in group III (1.330 MPa.m^1/2^) with the blending of the superior concentration of both silver and titanium nanoparticles, respectively. The results suggested that porcelain specimens increase in toughness after sintering in the presence of silver and silver nanoparticles, which was confirmed by the KIC toughness values.

Porcelain can be toughened by incorporation of fine particles of metals into the matrix. Toughening mechanisms act behind the crack tip to resist its opening. The lamella structures hold the fracture surfaces together after the crack has propagated through the matrix. Cracks and microcracking ‒ smaller cracks form in the material around the main crack ‒ relieve the stress at the crack tip by effectively increasing the material's compliance and transformation toughening.^17,18^

KIC values were significantly higher in group I (1.949 MPa.m^1/2^) and group II (2.002 MPa.m^1/2^) when compared to the control group (1.8087 MPa.m^1/2^). Incorporation of silver and titanium nanoparticles increased the fracture toughness of porcelain. Studies on titanium dioxide (TiO_2_) nanoparticles have documented increased hardness, ductility and yield strength. De la Fuente et al, Esparza-Vázquez et al, Siegel et al and Nazari et al^7,13,14,17^ found significant increases in toughness after incorporation of nanoparticles into ceramics. Titanium dioxide nanoparticles might be used in the form of microscale pigments or as nano-objects. ^20^

Silver oxide nanoparticles have unique optical, electrical and thermal properties and are incorporated into products that range from photovoltaics to biological and chemical.^21^ Mohsen et al^11^ and Pal et al ^22^  established the antibacterial activity of silver nanoparticles. They compared the effects of silver nanoparticles and silver hydroxyapatite nanoparticles on color and fracture strength of dental ceramic, where similar conclusions were elicited; incorporation of silver nanoparticles increased the fracture strength of dental ceramics, whereas incorporation of silver hydroxyapatite nanoparticles decreased the fracture strength of dental ceramic and both nanoparticles affected the color properties adversely, limiting the concentration of silver nanoparticles to 2 wt%.  Uno et al^1^ and Fujieda et al ^15^  reported improved fracture resistance after incorporation of silver nanoparticles. The investigation showed greater susceptibility to corrosion resistance and coefficient of thermal expansion.

The fracture toughness decreased in group III specimens (1.330 MPa.m^1/2^) in comparison to the control group. The network bonding effect of different metal nano-oxides (silver and titanium) can lead to the decreased strength, for small clusters. The dispersion of the nanoparticles was heterogeneous with large agglomerates in SEM images. The heterogeneous shape of the nanoparticles affects the volume of nano-layers surrounding the particles, leading to agglomeration affecting the strength of the material.^23^

This study showed improvements in fracture resistance of ceramic with the individual concentration of nanoparticles of silver and titanium.  In combination with highest percentage it decreased the strength. Further studies are required on different combinations or optimizations of silver and titanium nanoparticles and its influence on fracture toughness. Different combinations of composite mixture were not evaluated in this study, necessitating analysis in further studies.

## Conclusion

Within the limitations of this study, the fracture resistance decreased with the blending of superior concentration of silver and titanium nanoparticles obtained from reinforcements when compared with the conventional ceramics.

## Authors’ Contributions

VK was responsible for the design, analysis and writing the manuscript. NGC was responsible for the concept, design, expert opinion, analysis, interpretation and writing the manuscript. JRR prepared the manuscript. BM helped prepare the manuscript.

## Acknowledgments

The authors thank Prof. Dr. G. Rajkumar, Head of Department, Department of Physics, Eashwari Engineering College, Ramapuram, Chennai. India.

## Funding

No funding.

## Competing Interests

The authors declare no conflict(s) of interest related to the publication of this work.

## Ethics Approval

In vitro study approved by IRB. No human intervention.
